# Supplementary figures and images for: The effectiveness of Non-pharmaceutical interventions in reducing the COVID-19 contagion in the UK, an observational and modelling study
Source: PLoS One. 2021 Nov 29;16(11):e0260364. doi: 10.1371/journal.pone.0260364 (PMC8629270; doi:10.1371/journal.pone.0260364)

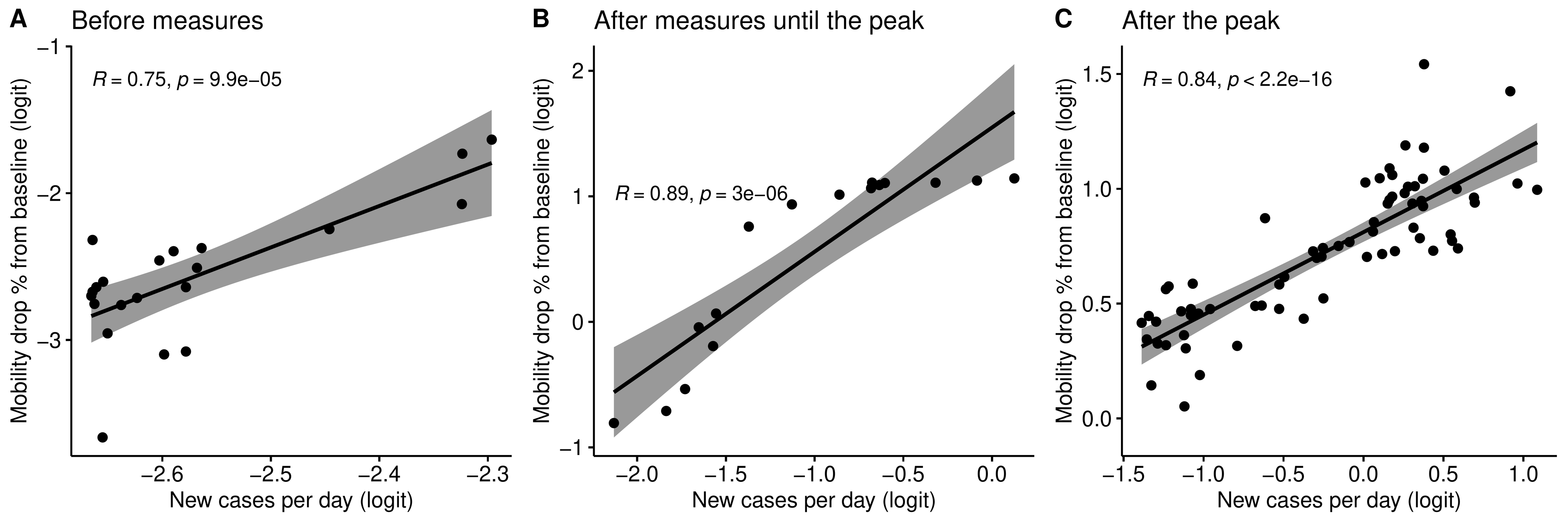

Supplement: S1 Fig — A regression line is drawn in black with the corresponding 95% confidence interval in grey. We also show the Pearson’s correlation coefficient—"R" and the corresponding p.value—"p" assessing the significance of the correlation coefficient estimate. A: The relationship before the advice for maintaining physical distancing. B: The relationship after the advice for physical distancing but before enforceable lockdown. C: The relationship after lockdown. (TIF) [file pone.0260364.s001.tif]
