# Supplementary material for: The effectiveness of Non-pharmaceutical interventions in reducing the COVID-19 contagion in the UK, an observational and modelling study
Source: PLoS One. 2021 Nov 29;16(11):e0260364. doi: 10.1371/journal.pone.0260364 (PMC8629270; doi:10.1371/journal.pone.0260364)
Supplement: Attachment — Submitted filename: Response to Reviewers.docx [file pone.0260364.s002.docx]

|   Georgios Baskozos,  West Wing, Level 6  John Radcliffe Hospital, Oxford, OX3 9DU  **www.ndcn.ox.ac.uk**  **Email**: georgios.baskozos@ndcn.ox.ac.uk |  |
| --- | --- |

Dear editors,

Thank you for giving us the opportunity to submit a revised manuscript to PLOS One. Below is a detailed response to all the points raised by the reviewer. Our point by point response is in **bold.** In addition to the points discussed below we have edited the manuscript to be fully concordant with the formatting templates provided, amended the funding statement to reflect all funding sources, uploaded the missing “Supporting Information Figure 1” and reviewed our reference list. We thank the editor for providing constructive feedback on this issues. We have taken all their comments into account and we feel that they made the manuscript stronger.

Yours sincerely,

Georgios Baskozos

Reviewer #1: The authors would need to revise the manuscript in line with the following;

Introduction:
The statement on the second paragraph may need to be revised to reflect the present day realities of availability of vaccines but with problems of vaccine hesitance.

**Thank you very much for this comment. The reviewer is right. We have changed this statement accordingly both in the introduction and the discussion.**

Methods:
The authors may need to state how the data from Google’s “COVID-19 Community Mobility Reports were validated and abstracted.

**We have added a short paragraph when we present Google’s “COVID-19 Community Mobility Reports” where we discuss the expected range of values, anonymisation and data aggregation. We believe this additional information shows how data were validate to be within the expected range and provided full details on data collection and aggregation.**

Discussion:
The discussion section will need to revised from just discussing the findings of this study solely to brining into context findings of others studies

**We have added a short paragraph which relates our work and results with other closely related studies and added the appropriate references. We have also moved part of our conclusion to the discussion section.**

Conclusion:
The authors may consider summarising the conclusion as it is too lengthy in this present form

**We have significantly shortened the conclusion.**
